# Spatiotemporal summation and correlation mimicked in a four-emitter light-induced artificial synapse

**DOI:** 10.1038/s41598-018-20595-8

**Published:** 2018-02-01

**Authors:** Zheng Shi, Shuai Zhang, Jialei Yuan, Bingcheng Zhu, Yuan Jiang, Xiangfei Shen, Yongjin Wang

**Affiliations:** Peter Grünberg Research Centre, Nanjing University of Posts and Telecommunications, Nanjing, 210003 China

## Abstract

In the brain, each postsynaptic neuron interconnects many presynaptic neurons and performs functions that are related to summation and recognition as well as correlation. Based on a convolution operation and nonlinear distortion function, we propose a mathematical model to explore the elementary synaptic mechanism. A four-emitter light-induced artificial synapse is implemented on an III-nitride-on-silicon platform to validate the device concept for emulating the synaptic behaviors of a biological synapse with multiple presynaptic inputs. In addition to a progressive increase in the amplitude of successive spatiotemporal excitatory postsynaptic voltages, the differences in the stimulations are remembered for signal recognition. When repetitive stimulations are simultaneously applied and last over a long period of time, resonant spatiotemporal correlation occurs because an association is formed between the presynaptic stimulations. Four resonant spatiotemporal correlations of each triple-stimulation combination are experimentally demonstrated and agree well with the simulation results. The repetitive stimulation combinations with prime number-based periods inherently exhibit the maximum capacity of resonant spatiotemporal correlation. Our work offers a new approach to building artificial synapse networks.

## Introduction

The human brain has approximately 100 billion biological neurons, and each neuron can form thousands of links with other neurons to sense stimulations and to pass signals to other neurons at the same time^1–4^. During synaptic transmission within the brain, the presynaptic neurons cause excitatory postsynaptic voltages (EPSVs) at the postsynaptic neurons, which are stored, encoded and identified for neural computing. If the stimulated signals are different, they can be memorized and identified through summed EPSVs, which leads to the coexistence of the dual functionalities of memory and recognition in the brain. According to Hebb’s theory^5,6^, neurons that fire together wire together. When repetitive spatial stimulations are active and last over a long period of time, there will be a long-lasting enhancement in memory and a stronger identification of stimulations. During these processes, coactive stimulations can associate with one another, and special correlations among them are formed, which can be observed from the integrated EPSV information. In particular, these presynaptic stimulations can be highly cooperative for diverse associations to produce different postsynaptic outputs, which are of great interest for a multifunctional biological nervous system.

In addition to directly studying synaptic transmission in the mammalian brain to explore these intriguing phenomena^7–11^, a variety of artificial synapses that are similar to the gap junction-based biological synapse have been demonstrated to mimic the biological nervous system for the further hardware implementation of artificial neural systems. Complementary metal-oxide semiconductor neurons have been adopted for the implementation of integrated neural networks to emulate synaptic functions such as training and spike timing-dependent plasticity^12^. Synaptic plasticity is mimicked in Ag_2_S or WO_x_-based inorganic synapses^13,14^. Laterally proton-coupled transistors have been used to build artificial synapse networks^15,16^. Tunable memristive phenomena have been demonstrated by MoS_2_-based memristors^17^. Carbon nanotube synapses have been reported to emulate the dynamic logic and learning functions^18,19^. Optical neuromorphic computing has been proposed using an on-chip nanophotonic system with a faster speed than electronic neural architectures^20^. In particular, many impulses are carried along the optic nerve fibers in the eye when we look at an object. The information is combined and transmitted to the brain. Finally, the brain interprets the information and recognizes what we see. In our previous work, we developed light-induced artificial synapses to emulate various summation behaviors^21–23^. Compared with synaptic electronics, a light-induced artificial synapse is analogous to the optic nerves for mimicking the sensation of sight. Based on the improved multistore memory model^24,25^, we propose a mathematical model to investigate the synaptic behaviors that are related to summation, recognition and resonant spatiotemporal correlation in the nervous system. The device concept is validated by a four-emitter light-induced artificial synapse, which can simultaneously mimic memory, recognition and correlation.

**Figure 1:**
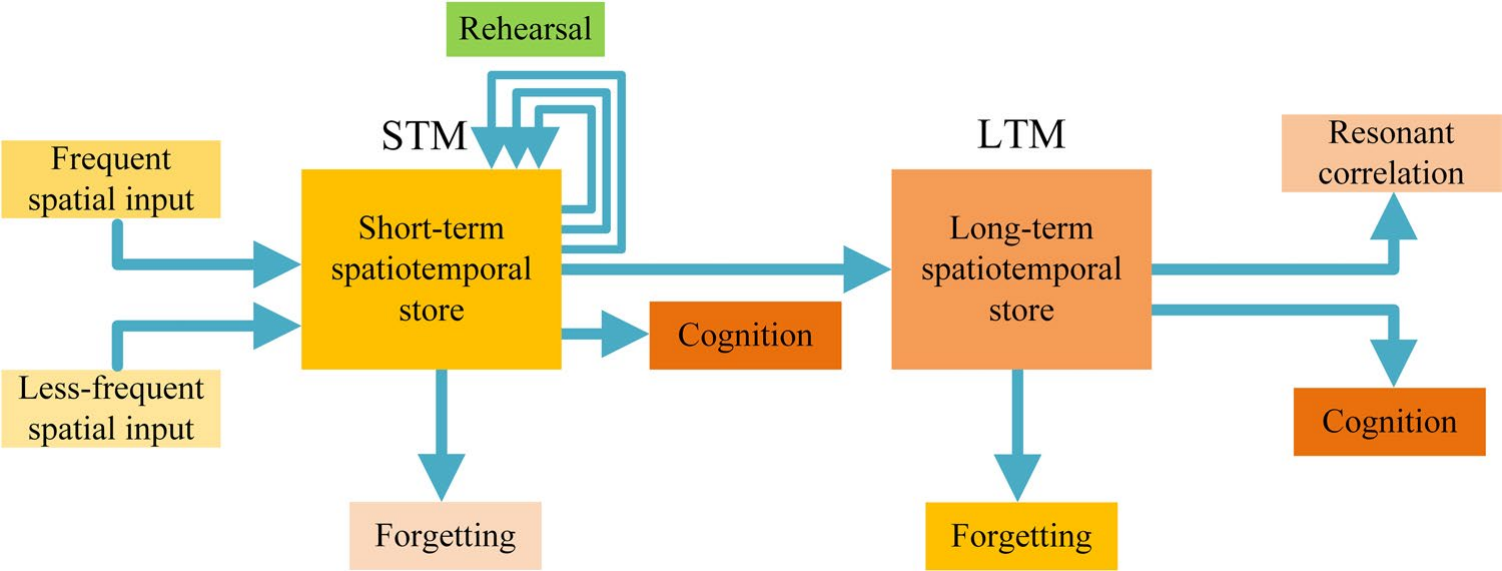
Schematic illustration of the psychological model for multiple spatiotemporal stimulations.

Figure 1 shows a schematic illustration of a psychological model for multiple spatiotemporal stimulations, which is inspired by the multistore memory model. Presynaptic stimulations are accumulated, encoded and stored in short-term memory (STM) for less-frequent spatial input, and the differences in the spatial stimulations can be identified for signal recognition. In the STM process, the generated EPSV amplitude is relatively small. Therefore, information will decay and be lost rapidly. Imagine a biological neuron that simultaneously receives enough repetitive stimulations from different terminals at the same time. In this case, repeated spatial rehearsal events are strongly active at periods small enough to cause temporal EPSV summation, and many synapses are stimulated simultaneously to cause significant spatial EPSV summation. The spatiotemporal EPSV summation is a long-term rehearsal process. Multiple repetitive presynaptic inputs will generate higher EPSV amplitudes, and the decay time is significantly elongated, which is beneficial to signal transmission. Thus, long-term memory (LTM) occurs because of spatiotemporal EPSV summation. The EPSV summation involves combining information from different presynaptic stimulations and can be interpreted for signal recognition due to the differences in the presynaptic inputs. Since repetitive stimulations are periodically applied, periodic postsynaptic outputs are generated. In addition to signal recognition, coactive stimulations can cooperate with one another to produce resonant spatiotemporal correlations during the LTM process when these repetitive stimulations last for a long time^5,26^.

## Results and Discussion

Based on the convolution operation and nonlinear distortion function, a mathematical model is constructed to investigate the resonant spatiotemporal correlation in the biological nervous system. The relationship between the presynaptic input *x*_*i*_*(t)* and the postsynaptic output *y(t)* can be expressed as

1 $$y(t)=f((\sum _{i=1}^{N}{x}_{i}(t)\ast h(t))$$

2 $$h(t)=\frac{t}{2{\sigma }_{1}^{2}}exp(-\frac{t}{2{\sigma }_{1}^{2}})+\frac{t}{2{\sigma }_{2}^{2}}exp(-\frac{t}{2{\sigma }_{2}^{2}})$$

where i = 1, 2, 3…; * denotes the convolution operation, which is defined as $f(t)\ast g(t)={\int }_{-\infty }^{\infty }f(\tau )g(t-\tau )d\tau$; and *h*(*t*) is a function that models the linear response of the channel, which can also be referred to as the impulse response function. The two parameters ${\sigma }_{1}^{2}$ and ${\sigma }_{2}^{2}$ can control the delay spread of the channel. Specifically, when ${\sigma }_{1}^{2}$ is large, the delay is large; the other parameter $\,{\sigma }_{2}^{2}$ is used to model the fast response of the channel; $f(\cdot )$ is a function that models the nonlinearity of the device, which is defined as

3 $$f(v)={V}_{max}(1-{e}^{-sv})$$

where *V*_*max*_ is the saturated output voltage of the device, and *s* is a parameter that determines the level of nonlinearity. Expanding the exponential component using a Taylor series, we obtain

4 $$f(v)={V}_{max}(-\sum _{n=1}^{\infty }\frac{{(-sv)}^{n}}{n!})$$

When *s* → 0, eq. (4) approaches a linear function, which is expressed as

5 $$f(v)=(s{V}_{max})v+o(s)$$

It can be observed that the postsynaptic output is a superposition of the total presynaptic inputs, where the distortion is modeled by the nonlinear function *f*(*v*) in (3).

**Figure 2:**
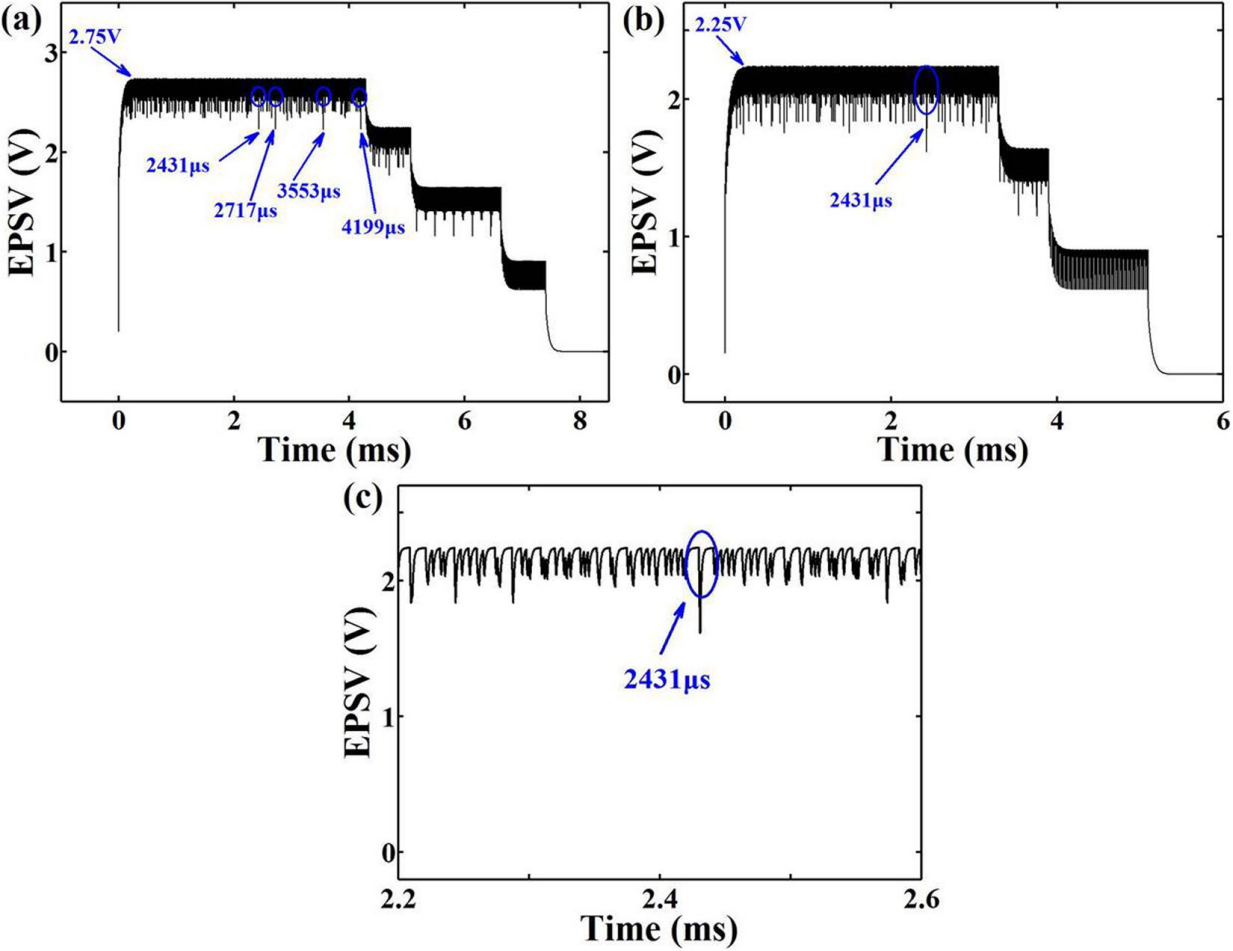
Calculated spatiotemporal EPSV summations: (**a**) four presynaptic inputs; (**b**) three presynaptic inputs; (**c**) the zoomed-in image of resonant EPSV summation at 2431 µs.

The repetitive stimulation combinations with prime number-based periods inherently exhibit the maximum capacity of resonant spatiotemporal correlation because the prime number has no positive divisors other than 1 and itself. Imagine a neuron that has a synaptic capacity of four synapses and simultaneously receives four repetitive stimulations with a period combination of (11 µs, 13 µs, 17 µs, 19 µs). The pulse intervals of repetitive stimulations are 1 µs, and the numbers of stimulations are 390 to generate the effective temporal EPSV summation. Figure 2(a) shows the calculated spatiotemporal EPSV summation. Because of the spatial EPSV summation, the saturated EPSV amplitude is increased from 2.25 V to 2.75 V in comparison with that of the three repetitive stimulation combinations of (11 µs, 13 µs, 17 µs), as illustrated in Fig. 2(b). Coactive stimulations can cooperate to produce associations among these periodic stimulations when the repetitive stimulations are periodically applied and last over a long period of time. The generated postsynaptic outputs could have the same period, which would lead to resonant correlations. Thus, resonant spatiotemporal correlations among the four repetitive stimulations occur as well as synaptic strengthening in the EPSV amplitude during the LTM process. There are four resonant correlated combinations when the triple stimulations are highly correlated with respect to one another. Three repetitive stimulations of (11 µs, 13 µs, 17 µs), (11 µs, 13 µs, 19 µs), (11 µs, 17 µs, 19 µs), and (13 µs, 17 µs, 19 µs) occur at the same time. The resonant periods are 2431, 2717, 3553 and 4199 µs, respectively. Figure 2(c) shows a zoomed-in image of resonant EPSV summation at 2431 µs, where three stimulations of (11 µs, 13 µs, 17 µs) occur at the same time with a resonant period. These results indicate that the postsynaptic neuron requires a longer time to form the correlations among the presynaptic stimulations when more presynaptic neurons simultaneously participate in the stimulation process.

**Figure 3:**
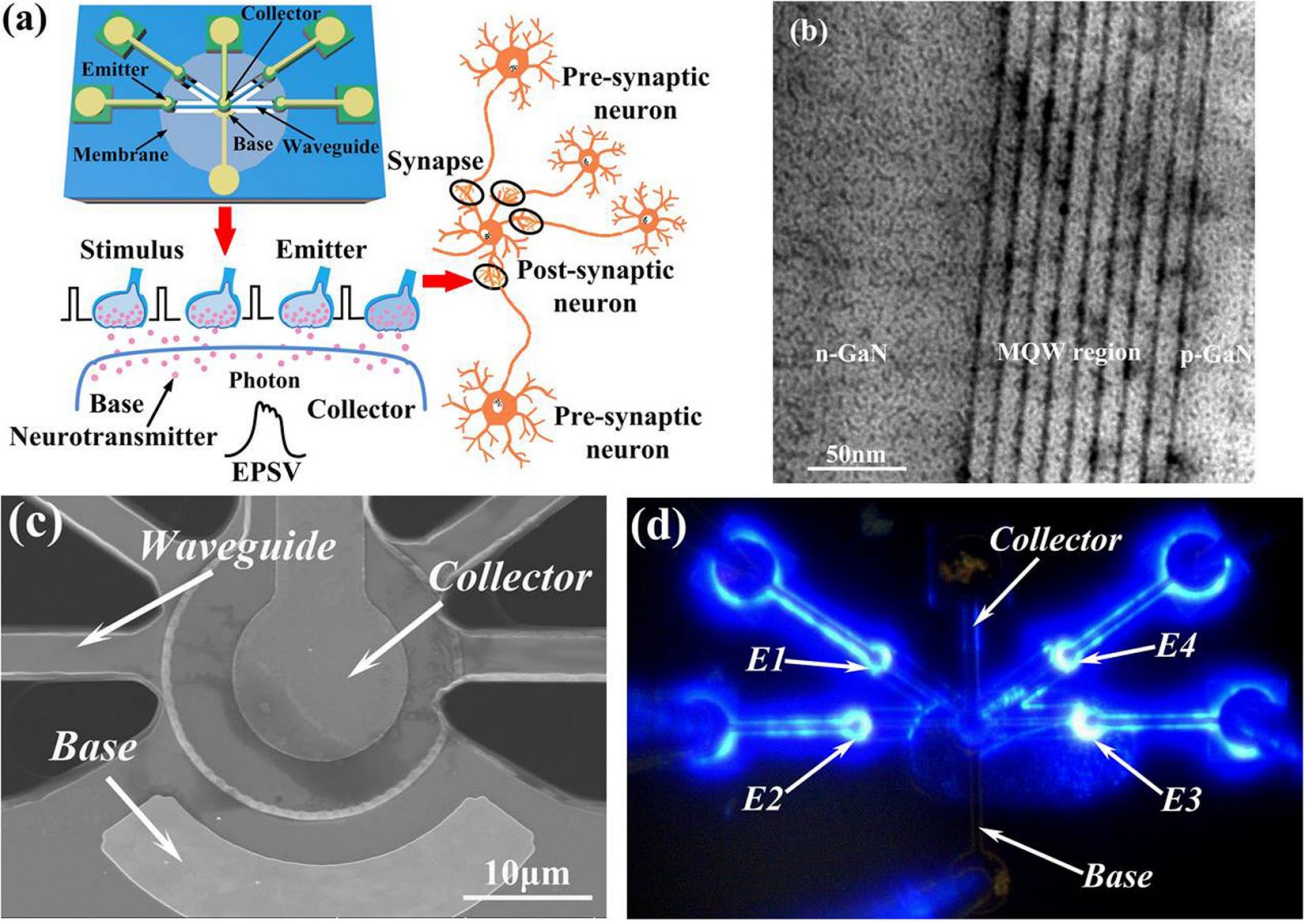
(**a**) Schematic of a four-emitter light-induced artificial synapse. (**b**) Cross-sectional TEM image of the InGaN/GaN MQW structure. (**c**) SEM image of the fabricated artificial synapse. (**d**) Light emission of the four-emitter artificial synapse at the injection currents of 100 µA.

A four-emitter light-induced artificial synapse is proposed to validate the synaptic device concept, as schematically illustrated in Fig. 3(a). The artificial synapse is a six-terminal device, which consists of four MQW-emitters (E), one MQW-collector (C) and one base (B). The emitters are adopted as presynaptic terminals, and the collector is used as a postsynaptic neuron. The stimulated light from the emitter will cause a light-induced voltage at the collector, which is similar to the voltage-gated ion channel in the biological synapse. The artificial synapse is implemented on a 2-inch III-nitride-on-silicon wafer^27^. The top epitaxial structures comprise a nine-period multiple quantum well (MQW) active region with 3-nm-wide InGaN QWs and 10-nm-wide GaN barriers, as shown in Fig. 3(b). The carrier concentrations of the Si-doped n-GaN and Mg-doped p-GaN layers are approximately 8 × 10^18^ and 2.0 × 10^20^ cm^−3^, respectively, which leads to the formation of a MQW-based p-n junction diode. Because the InGaN/GaN MQW-diode has selectable functionalities as an emitter or detector, both the MQW-emitter and the MQW-collector are fabricated on the same membrane, sharing an identical MQW-diode structure. Figure 3(c) shows a magnified scanning electron microscope (SEM) image of the artificial synapse. Four 100-µm-long and 6-µm-wide suspended waveguides impinge onto the MQW-collector to guide the emitted light from the MQW-emitter. The MQW-collector absorbs the in-plane guided light to generate a spatiotemporal EPSV summation when the MQW-emitters are biased to generate light. Figure 3(d) shows the simultaneous light emission of four emitters at injection currents of 100 µA. Four emitters are electrically isolated from the collector and can independently emit light^28,29^.

**Figure 4:**
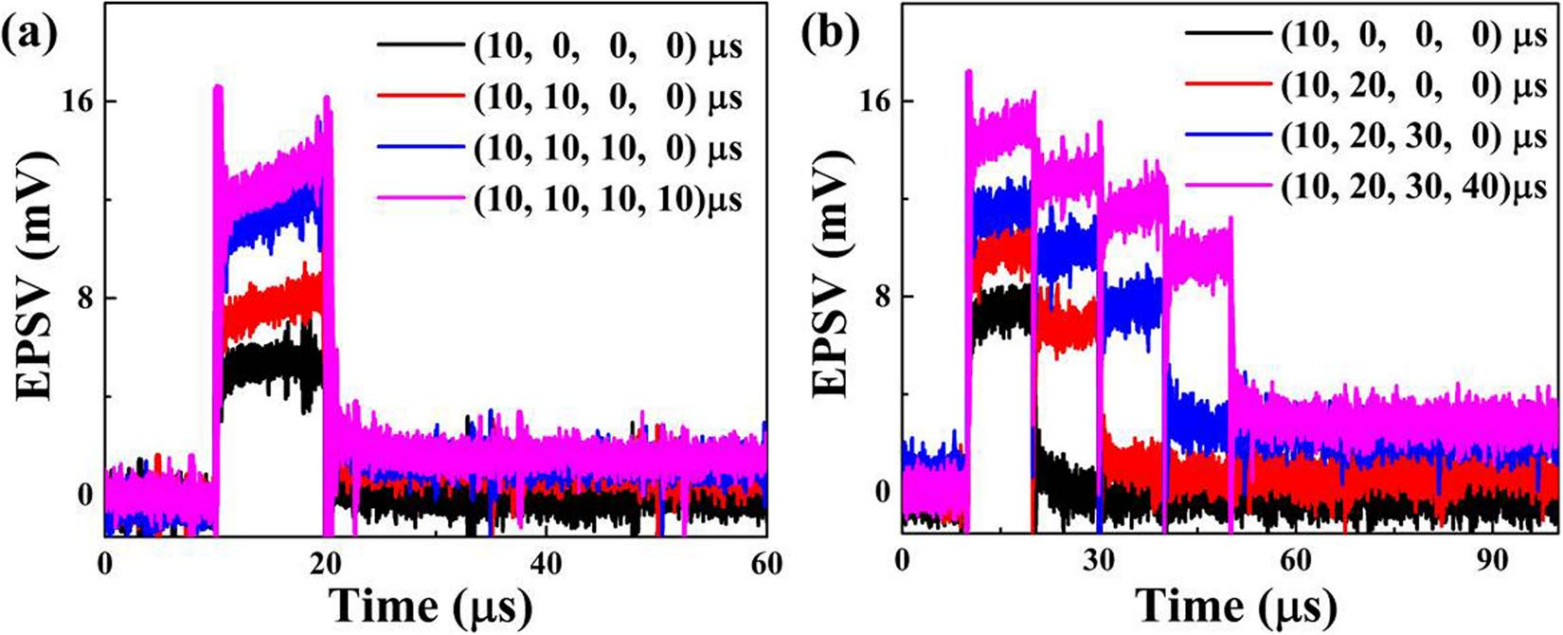
(**a**) Spatial EPSV summation as a function of the number of stimulations. (**b**) Spatial EPSV summation versus different duration combinations of stimulation.

Spatial EPSV summation occurs when a biological neuron receives inputs from multiple presynaptic terminals at the same time, which is experimentally mimicked by the fabricated four-emitter artificial synapse. The MQW-emitters as the presynaptic terminals are simultaneously biased by two pulse function arbitrary generators to generate the pulse illuminations, and spatial EPSV summation occurs when the MQW-collector as the postsynaptic receptor absorbs the in-plane accumulated light from suspended waveguides. Figure 4(a) shows the spatial EPSV summation versus the number of stimulations, in which the duration of the stimulation is 10 µs. Because the generated EPSV amplitude depends on the light absorption by the MQW-collector, a progressive increase in the amplitude of the summed EPSV is observed with a prolonged decay time as the number of stimulations is increased from 1 to 4. The signal differences can be identified from the spatial EPSV summation when the duration of stimulation is different. Figure 4(b) demonstrates the spatial EPSV summation as a function of different durations of combinations of stimulation. Compared to a single stimulation with a duration of 10 µs, the summed EPSV amplitudes are increased for the duration combinations of (10 µs, 20 µs), (10 µs, 20 µs, 30 µs) and (10 µs, 20 µs, 30 µs, 40 µs). Distinct changes are observed in the spatial EPSV summation when stimulations with shorter durations are completed. Coactive presynaptic terminals lead to a strengthened synapse, which can store and identify the signal differences through spatial EPSV summation. The coexistence of memory storage and recognition plays an important role in STM for a biological neuron system with multiple presynaptic inputs.

**Figure 5:**
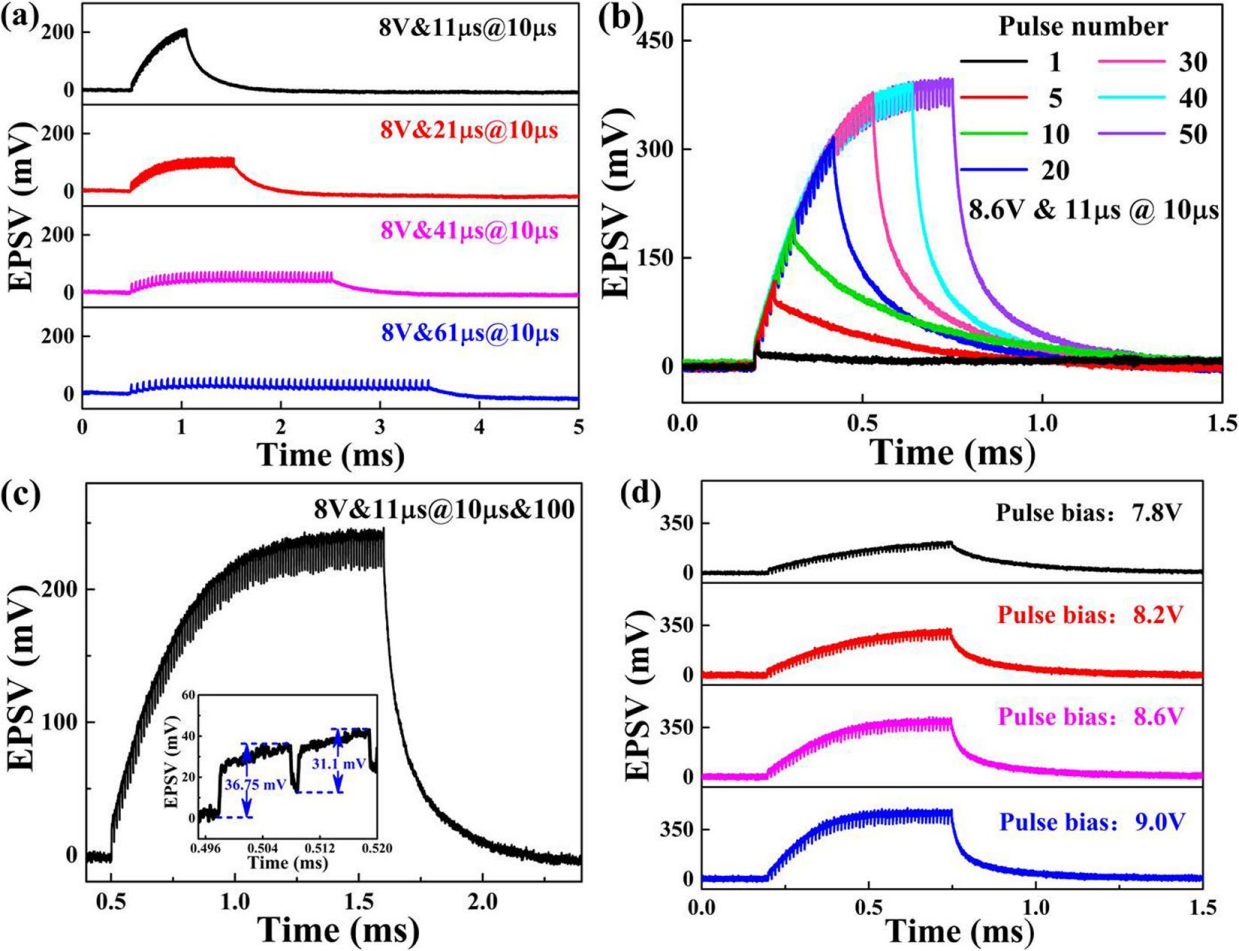
(**a**) Temporal EPSV summation as a function of the period of repetitive stimulation. (**b**) Temporal EPSV summation versus the number of repetitive stimulations. (**c**) Trade-off between the EPSV summation and habituation. (**b**) Temporal EPSV summation as a function of the amplitude of repetitive stimulation.

A temporal EPSV summation is produced when the repetitive stimulations are active for sufficiently small periods of time. Figure 5(a) shows the temporal EPSV summation at the MQW-collector as a function of the period of repetitive stimulation by a single MQW-emitter, in which the MQW-emitter is pulse-biased at 8.0 V to generate the stimulation with a duration of 10 µs and the number of repetitive stimulation is fixed at 50. The temporal EPSV summation is significantly reinforced as the repetitive stimulation period decreases from 61 µs to 11 µs with a distinct prolongation of the decay time. Figure 5(b) shows the temporal EPSV summation as a function of the number of repetitive stimulations. The MQW-emitter is biased at 8.0 V to generate the pulse stimulation, in which the duration of stimulation is 10 µs and the period of repetitive stimulation is fixed at 11 µs. During the repetitive stimulation process, each individual pulse contributes a residual facilitation to form the total facilitation, which leads to an increased EPSV amplitude. On the other hand, repeated stimulation gives rise to a progressively smaller EPSV amplitude due to habituation, as shown in the inset of Fig. 5(c). As a result, the summed EPSV amplitude is first enhanced and eventually saturated as the number of repetitive stimulation increases, which indicates that the EPSV amplitude caused by the initial stimulation has a significant influence on the saturated EPSV amplitude. Figure 5(d) shows the temporal EPSV summation as a function of the amplitude of repetitive stimulation, in which the duration of stimulation is 10 µs, the period of repetitive stimulation is fixed at 11 µs, and the number of repetitive stimulations is fixed at 10. In comparison with the pulse bias of 7.8 V, the MQW-emitter biased at 9.0 V will generate more photons, which results in a higher amplitude of the stimulation. It is apparent that a higher amplitude of the initial stimulation leads to a higher saturated EPSV amplitude and reaches the saturated EPSV amplitude with a smaller number of stimulations. When these spatiotemporal effects occur, the EPSV values are integrated to a higher amplitude. If the EPSV threshold value is set in an artificial neural network (ANN) system, the EPSV value generated by these spatiotemporal effects would exceed the threshold value. A decision process is made, and the generated EPSV information is thus stored to trigger an output, which can be transmitted to an external memory circuit. The information can be further interpreted for real neuromorphic computing hardware.

**Figure 6:**
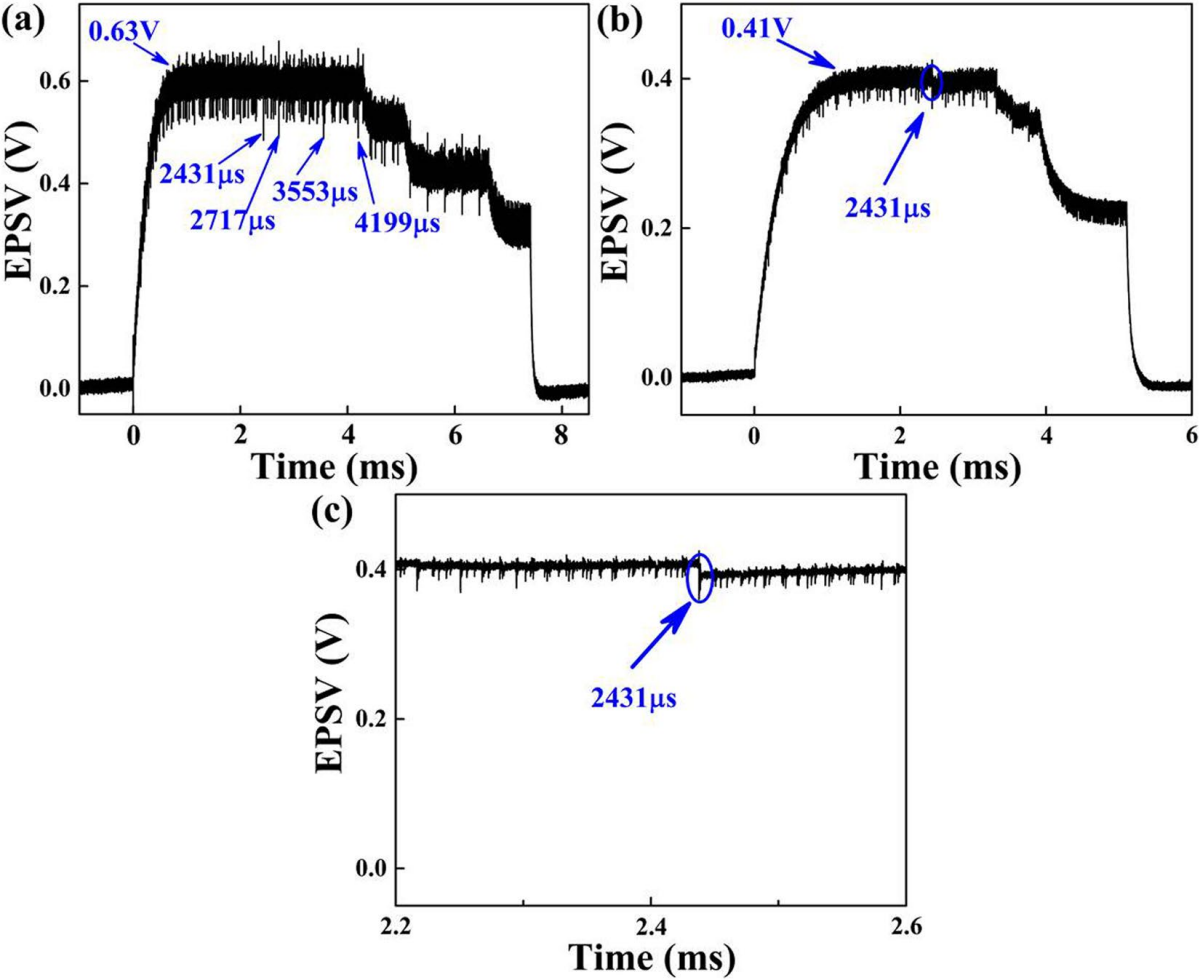
Spatiotemporal EPSV summations: (**a**) four presynaptic inputs; (**b**) three presynaptic inputs; (**c**) the zoom-in image of resonant EPSV summation at 2431 µs.

Figure 6(a) shows the measured spatiotemporal EPSV summation of four repetitive stimulation combinations (11 µs, 13 µs, 17 µs, 19 µs), in which the same stimulation numbers are 390 and the same intervals of repetitive stimulations are 1 µs. The spatiotemporal correlation of each of the triple-repetitive stimulations are experimentally demonstrated with resonant periods of 2431, 2717, 3553 and 4199 µs, respectively. The saturated EPSV amplitude is increased from 0.41 V to 0.63 V in comparison with that of three repetitive stimulation combinations (11 µs, 13 µs, 17 µs), as illustrated in Fig. 6(b). The resonant EPSV summation is magnified in Fig. 6(c), where three stimulations of (11 µs, 13 µs, 17 µs) occurs simultaneously with a resonant period of 2431 µs. The spatiotemporal correlations of each of the dual-repetitive stimulations are observed with resonant periods of 143, 187 and 221 µs. These experimental results agree well with the simulation results, which indicates that long-term spatiotemporal EPSV summation can give rise to resonant spatiotemporal correlations among multiple presynaptic inputs for complex neural function.

The four-emitter artificial synapse yields STM in response to less-frequent spatial stimulations. The differences in the spatial stimulations can be remembered for signal recognition. In response to frequent spatial stimulations, the artificial synapse yields LTM by causing spatiotemporal EPSV summation, in which recognition as well as resonant correlations are formed. When the EPSV threshold is set, the EPSV generated by these spatiotemporal effects can be integrated into a larger and more complex ANN system, in which decision, memory and learning processes are performed. Both experimental results and mathematical simulations are promising for the exploration of the elementary synaptic mechanism for artificial neural networks.

## Methods

### Fabrication

The top III-nitride layers are grown on (111) silicon substrate by metal organic chemical vapor deposition. The starting III-nitride-on-silicon wafer is first patterned by photolithography and formed by induced coupled plasma reactive ion etching (ICP-RIE) of III-nitride epitaxial films using Cl_2_ and BCl_3_ hybrid gases with the etching rate of ~120 nm/min. In association with lift-off and rapid thermal annealing in an N_2_ atmosphere, the 20 nm/180 nm Ni/Au metal stacks are evaporated as p- and n-electrodes. Then, waveguide structures are defined by photolithography and etched with a depth of ~3 μm by ICP-RIE, where thick AZ4620 photoresist is used as the etching hard mask. After protecting the top device structure with thick AZ4620 photoresist, deep reactive ion etching is conducted to remove ~195-μm-thick silicon substrate, in which alternating steps of SF_6_ etching and C_4_F_8_/O_2_ passivation are adopted. Subsequently, the underlying layer of the suspended waveguide is etched away without an additional etching hard mask by ICP-RIE backside thinning of the III-nitride films, which leads to an ultrathin membrane-type device architecture.

### Optical characterization

The fabricated four-emitter artificial synapse is mounted on a Cascade PM5 probe station and is probed by six dc probes. Two Keysight 81160 A pulse function arbitrary generators are used to control four MQW-emitters to output light synchronously, and thus, the MQW-collector absorbs the in-plane light at the same time. The generated spatiotemporal EPSV summations are directly characterized by an Agilent DSO9254A digital storage oscilloscope.
